# osl-ephys: a Python toolbox for the analysis of electrophysiology data

**DOI:** 10.3389/fnins.2025.1522675

**Published:** 2025-02-21

**Authors:** Mats W. J. van Es, Chetan Gohil, Andrew J. Quinn, Mark W. Woolrich

**Affiliations:** ^1^Oxford Centre for Human Brain Activity, Wellcome Centre for Integrative Neuroimaging, Department of Psychiatry, University of Oxford, Oxford, United Kingdom; ^2^Brain and Mind Centre, School of Medical Sciences, University of Sydney, Sydney, NSW, Australia; ^3^Centre for Human Brain Health, School of Psychology, University of Birmingham, Birmingham, United Kingdom

**Keywords:** magnetoencephalography (MEG), electroencephalography (EEG), MNE-Python, electrophysiology, python, toolbox, analysis, M/EEG

## Abstract

We describe OHBA Software Library for the analysis of electrophysiology data (osl-ephys). This toolbox builds on top of the widely used MNE-Python package and provides unique analysis tools for magneto−/electro-encephalography (M/EEG) sensor and source space analysis, which can be used modularly. In particular, it facilitates processing large amounts of data using batch parallel processing, with high standards for reproducibility through a config API and log keeping, and efficient quality assurance by producing HTML processing reports. It also provides new functionality for doing coregistration, source reconstruction and parcellation in volumetric space, allowing for an alternative pipeline that avoids the need for surface-based processing, e.g., through the use of Fieldtrip. Here, we introduce osl-ephys by presenting examples applied to a publicly available M/EEG data (the multimodal faces dataset). osl-ephys is open-source software distributed on the Apache License and available as a Python package through PyPi and GitHub.

## 1 Introduction

The analysis of neuroimaging data typically involves a series of complicated analysis steps which are deployed heterogeneously to suit both the dataset and the scientific question. In non-invasive human electrophysiology data, particularly magnetoencephalography (MEG) and electroencephalography (EEG), these steps include but are not limited to: preprocessing to clean the raw recordings, co-registration with other data modalities (e.g., Polhemus, MRI), source reconstruction, and a plethora of (mass) univariate or multivariate statistical analyses.

To this aim, the field has traditionally relied on a suite of open-source software toolboxes developed by individual research groups or community efforts (Delorme and Makeig, 2004; Litvak et al., 2011; Oostenveld et al., 2011; Tadel et al., 2011; Jenkinson et al., 2012; Gramfort, 2013; Lopez-Calderon and Luck, 2014; OHBA Analysis Group, 2014). However, most of these rely on licensed, third-party software like MATLAB (The MathWorks Inc, 2020), which makes it costly and limits processing of large amounts of data. Because of this, there is a current shift in the field to adopt the Python programming language and multiple Python packages specifically designed for the analysis of electrophysiology data have recently been published (Gramfort, 2013; Schirrmeister et al., 2017; Lu, 2020; Sabbagh et al., 2020; Brodbeck et al., 2023; Gohil et al., 2023; Jas et al., 2023; Ågren, 2023). Of these, MNE-Python (Gramfort, 2013; Larson et al., 2023) is by far the most widely adopted.

The field also sees an increasing use of publicly available and large datasets. At the same time, there are higher requirements for transparency and reproducibility by academic journals. Therefore, analysis tools must adapt to help meet these needs. One solution is MNE-Python’s MNE-BIDS-pipeline, which offers automated processing of M/EEG data in BIDS format through a configuration API. However, specification of the configuration is arguably non-trivial, and the pipeline does not allow for full analysis flexibility.

Here, we present the osl-ephys toolbox, a free and open-source Python package for the analysis of electrophysiology data, and part of the *OHBA Software Library (OSL).* It is built on, and augments, the MNE-Python toolbox (Gramfort, 2013; Larson et al., 2023), and is developed with the following core principles:

1. Efficient processing of large amounts of data;
2. A concise *configuration* API that summarises a reproducible processing pipeline, and is easy to specify, interpret, and share;
3. Automatic generation of log files and HTML processing reports to enable reproducibility and provide quality assurance.
4. A modular setup, to facilitate integration with MNE-Python and other, third-party toolboxes in Python, or other programming languages (e.g., MATLAB).

Moreover, the toolbox contains unique processing functions for analysing M/EEG data, including FSL-based (Freesurfer independent) coregistration and volumetric source reconstruction pipeline (Jenkinson et al., 2012).

The toolbox has a user friendly Application Programming Interface (API) based on the specification of a “config” object, which concisely holds all the information needed for the processing steps to apply. This helps ensure reproducibility and facilitates the processing of large amounts of data. Below we briefly outline the architecture of the toolbox and the API, before presenting examples of each module.

## 2 Methods

### 2.1 Documentation

Documentation is available on readthedocs.^1^ This includes installation instructions (including setting up, e.g., Conda, FSL, etc.), a full list of function references (API), and tutorials. Source code is available on GitHub,^2^ and includes a list of requirements.

### 2.2 Overview

Osl-ephys uses MNE-Python as a backbone. For example, data classes like Raw, Epochs, Evoked, Info, and other classes are directly adopted and used, and M/EEG data derivatives are typically saved as “fif” files. This allows for a seamless integration of our toolbox with MNE-Python, or with other Python and MATLAB based toolboxes. It is straightforward for a user to a subset of the data processing pipeline in MNE-Python or other analysis toolboxes, while switching to and from ours. Table 1 summarises the added functionality of our toolbox over MNE-Python and MNE-BIDS-pipeline.

**Table 1:** Comparison of Osl-ephys to MNE-Python and MNE-BIDS-pipeline.

	Osl-ephys	MNE-Python	MNE-BIDS-pipeline
Pipeline API	Text config	Custom scripts	Text config
Flexibility – order of analysis steps	Any	Any	Fixed
Flexibility – third-party functions	Internal + external	Internal + external	Internal
Flexibility – input data organisation	Any format	Any format	BIDS format
Parallelisation Processing duration (Example in 3.1 or equivalent)	Parallelization using Dask 21 min on 16 CPU cores*	N/A 175 min on 1 CPU core*	Parallelization using Dask N/A
Transparancy/Reproducibility	Automatic generation of log files and HTML reports Config is saved in MNE objects	N/A	Automatic generation of HTML reports
Quality assurance	Automatic HTML subject and summary reports	Manual HTML report building (i.e., mne.Report)	Automatic HTML reports
Inputs for source reconstruction	FreeSurfer or FSL	FreeSurfer	FreeSurfer
Extra functionalities (not exhaustive)	Efficient interactive ICA labelling More options for detecting bad segments/channels FSL based coregistration and source reconstruction Volumetric parcellations Resolving sign ambiguity Orthogonalising parcel time courses GLM		

*Using Intel Xeon Gold 6,136 @ 3.0GHz processors and a total of 1.5 TB RAM.

Because the field of cognitive neuroscience is inherently multidisciplinary, individual researchers often lack formal training in programming. Therefore, usability and reproducibility are at the heart of the toolbox’s design philosophy. In particular, in the two main modules (preprocessing and source_recon, see below) a processing pipeline is defined in the form of a comprehensive configuration (“config”) API. This is a Python dictionary that specifies the call to individual functions and the parameter settings for each. The user will typically specify the config as a string. During the processing, the toolbox handles data bookkeeping and other complexities behind the scenes. A feature of the config is that it is easily shareable and can be easily used to reproduce analyses.

In the two main modules, the user typically interacts with high level pipeline “chain” and “batch” functions. These functions simply loop over the analysis steps specified in the config and call the appropriate function, together with the parameter arguments, to the data. Here, the batch function is used if the user wants to process multiple datasets at a time by efficiently looping the chain function over the datasets (see Section “Batch processing”). Both functions take as main input arguments the config, and the input and output directories. In addition, these functions can create log files and HTML reports (see below). The log files improve reproducibility by writing information on processing steps, random seed, etc. The reports summarise the processing and can guide quality assurance.

### 2.3 Modules

The toolbox contains several *modules* that provide tools for a specific analysis goal, e.g., preprocessing, source reconstruction, GLM, etc. These modules are designed such that they can be flexibly combined with third party toolboxes.

#### 2.3.1 Preprocessing

The preprocessing module contains functionality to pre-process M/EEG data. A simple example is given in Listing 1A. Preprocessing can include (wrapper) functions for, e.g., filtering, selecting data, resampling, bad channel/segment detection (Independent Component Analysis). It can also include more advanced analysis steps, e.g., spectral analysis using multi-tapers, and even group-level analysis. Beside the plethora of functions offered by MNE-Python, this module also contains unique functions, e.g., identifying bad channels/segments using a generalised ESD test (Rosner, 1983). It also includes wrappers to licensed MaxFilter™ software for maxwell filtering (though the open-source implementation in MNE-Python is also available). User defined functions can also be supplied to the toolbox to offer fully flexible pipelines (see Listing 1B and section “Custom functions”). Lastly, this module also allows for functions to be run on the group level, e.g., for statistical analysis.

**LISTING 1A:**
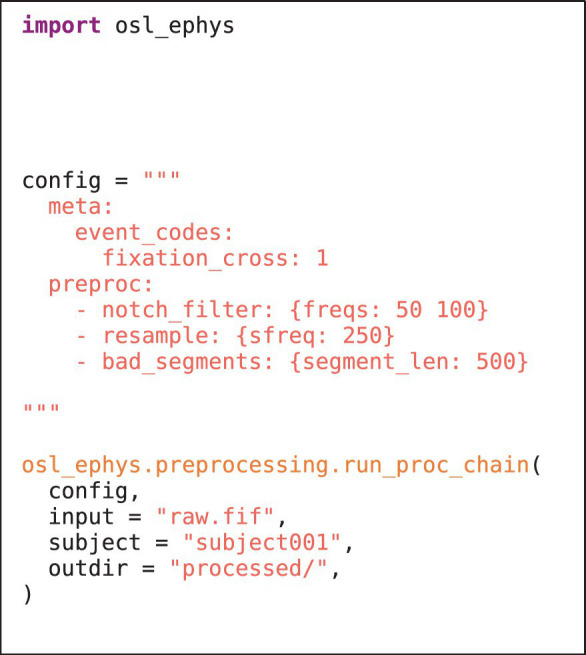
Example API in the preprocessing module. The config specifies the processing recipe applied to the input. Preprocessed data are saved in the processed/subject001 directory. Note that the source reconstruction module works similarly (see section “Examples”).

**LISTING 1B:**
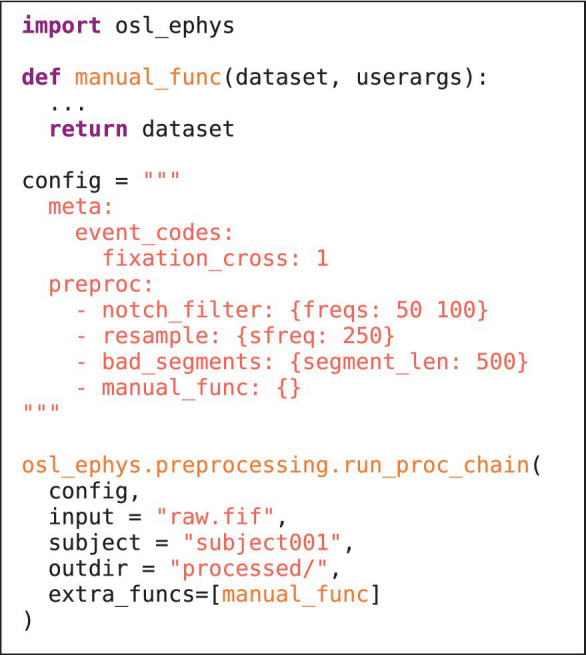
As in 1a, but now including a custom written function. The function is defined at the top, and takes as input “dataset” (a dictionary containing the MNE-Python objects, e.g., Raw), and a “userargs” dictionary (containing the function variable specifications). The function is included in the config, and supplied to chain function as a list of extra functions in the “extra_funcs” input variable.

The main user functions in the preprocessing module are the “chain” and “batch” pipeline functions. These load in the input data and represent them in a Python dictionary. Other derivatives generated during preprocessing will be added to the dictionary, which is ultimately returned by the *chain*/*batch* functions and individual items are saved to disk.

In addition to the above, the preprocessing module contains command line functionality for interactive labelling of bad ICs using a graphical user interface (see section “Examples”).

#### 2.3.2 Source reconstruction

The source_recon module contains tools for coregistration, volumetric and surface based source reconstruction, and for working with source space data more generally. The module enables a cortical surface source reconstruction pipeline based on FreeSurfer (Fischl, 2012), as is used in MNE-Python, or a volumetric source reconstruction pipeline based on FSL (Jenkinson et al., 2012), which is computationally less expensive. The FSL-based pipeline is currently not available in MNE-Python and is unique to our Python toolbox (though it still relies on low-level MNE-Python functions on the backend).

Typically, the steps used in the coregistration and source reconstruction pipeline are as follows:

1. Compute surfaces. Options for using FSL to extract the inner skull, outer skin (scalp) and brain surfaces from structural, T1 weighted MRI (sMRI) data, or to use FreeSurfer’s recon-all for cortical reconstruction.
2. Coregistration. Coregisters the M/EEG data, head digitisation points (i.e., Polhemus), and sMRI data. This can be done using FSL-based *Registration using Headshapes Including Nose in OSL* (RHINO), analogous to the MATLAB implementation (OHBA Analysis Group, 2014), or using the FreeSurfer-based automatic pipeline in MNE-Python.
3. Forward modelling. This is a wrapper for mne.make_forward_solution.
4. Source modelling. Typically done using a LCMV beamformer (Van Veen et al., 1997), or linear inverse methods (e.g., Minimum Norm Estimate).

Source reconstruction to the source dipole grid can then be followed by use of a pre-defined parcellation to extract parcel time courses, and including the reduction of spatial leakage and correction for sign ambiguities:

1. Parcellation. Parcellates a volumetric or surface-based dipole grid of source estimates by taking the principal component (or spatial basis set) of all dipoles in a parcel from a chosen Nifti parcellation or FreeSurfer annotation. Multiple standard volumetric parcellation templates are supplied [e.g., AAL (Tzourio-Mazoyer et al., 2002)], as well as fMRI-derived parcellations optimised for M/EEG data (i.e., with a lower amount of parcels to match the rank of M/EEG data; e.g., Giles39 (Colclough et al., 2015), and Glasser52 (Kohl et al., 2023)), as well as those supplied by FreeSurfer (e.g., Desikan et al., 2006; Destrieux et al., 2010; Thomas Yeo et al., 2011).
2. Symmetric orthogonalisation. The inverse problem in MEG is ill-posed because there are many more potential sources than MEG sensors. Estimated source activity is therefore correlated over spaces, which can cause spurious correlations between source time courses. We use symmetric multivariate leakage reduction (Colclough et al., 2015) to correct for these artificial correlations between a set of multiple regions of interest (i.e., parcels). Note that by being multivariate, this also corrects for so-called “ghost interactions” or “inherited connections” (Colclough et al., 2015; Palva et al., 2018).
3. Sign flipping. Ambiguities in the orientation of source dipoles and calculation of parcel time courses (e.g., through PCA) result in an ambiguity in the orientation, or polarity, of the parcel time courses. We adjust the orientation/polarity of the parcel time courses using the assumption that we expect correspondence in the functional connectomes from different subjects. As such, we maximise the correlation of the covariance matrices between subjects.

As with the preprocessing module, the source_recon module has high-level pipeline functions that work with a config API, and can optionally generate log files and HTML reports.

#### 2.3.3 Custom functions

It is in general possible to supply the chain and batch functions with custom written functions, defined by the user. These can be readily supplied to the toolbox by specifying the function in the config and supplying the list of custom functions to the extra_funcs input variable in the chain/batch function. The only requirement is that they adhere to the same structure as is used in lower level functions throughout the toolbox (this is slightly different for preprocessing and source reconstruction modules). This also facilitates external contributions to the toolbox: if a user defines a custom function that would be useful to a wider audience, it can easily be adopted into the main toolbox (i.e., through Github). The general function structure is outlined below.

*Preprocessing module*

- The function must take “dataset” and “userargs” as inputs
- Any options for the functions that are specified in the config can be retrieved from userargs [i.e., using userargs.pop()]
- The function must return “dataset”
- Any key in “dataset” can be manipulated, either in place or by adding a new key. New keys are saved by default in the subject folder.

*Source reconstruction module*

- The function can take any user-defined variables as input. All inputs to the pipeline function are always passed to the custom function.
- Changes must be directly saved to disk, rather than returning function outputs.

#### 2.3.4 General linear model (GLM)

The GLM module contains data classes and functions that combine functions from MNE-Python, custom code, and Python packages for linear modelling (Quinn, 2019; Quinn and Hymers, 2020) into modality specific (e.g., M/EEG data) tools for linear modelling. This includes the ability to do confound modelling and hierarchical modelling, including for spectral analysis [i.e., instead of the commonly used Welch periodogram, where the average is used; (Quinn et al., 2024)], and significance testing via non-parametric statistics. Additionally, it contains functions for visualising (statistically significant) effects.

The GLM functionality cannot be directly added to a config for the preprocessing or source_recon pipeline function, because of the added complexity of specifying a design matrix. These tools are typically applied in a separate Python script or by specifying a custom function that is supplied to the preprocessing config (see section “Examples”).

#### 2.3.5 Utilities

The utils module contains several helpful utility functions that can be directly deployed by the user, and/or is used in some of the higher level toolbox functions. Current utility functions include data loaders (for, e.g., OPM-MEG, SPM data), and functions for parallel processing (see *2.3 Batch processing*), logging, simulation, and file handling (see section “Examples”).

#### 2.3.6 Report

The report module allows for the generation of interactive HTML reports to gain insights into the analysis carried out by the high-level pipeline functions and guide quality assurance (QA). Separate reports are generated by default when using the preproc and source_recon *batch* processing functions, but can also be created manually. They include a subject report for in depth information about individual subjects/session, and a summary report summarising measures all across subjects/session.

The subject report contains information about each recording (e.g., number of sensors recorded, duration of the recording), and the analysis in the form of data tables and (interactive) figures, e.g., showing which channels were marked as “bad,” the result of the coregistration, etc. These figures are generated in a *reports* directory, in subdirectories for each individual subject/session, together with a data.pkl file. This file contains symbolic links to the appropriate figures, as well as plain text and numeric information about the session (e.g., also including a copy of the text from the log files).

The summary report gives an overview of the processing pipeline, including custom function definitions, and has interactive data tables summarising various metrics from all subject/session reports. This can guide the user to have a detailed look at specific subject report, for example for those subjects that had excessive number of channels marked as “bad,” or high errors in the coregistration.

### 2.4 Batch processing

Essential in the design philosophy is the ability to process large batches of data efficiently. The toolbox’s high-level “chain” functions therefore have “batch” function counterparts, which take in a config and lists of file paths for the data to be processed. We integrated Dask (Dask Development Team, 2016) for parallel processing these batches of data efficiently, using as many computational resources (i.e., CPU cores) as the user has available. While this parallelisation is also available in MNE-BIDS-pipeline, the analysis flexibility and unique config API are not.

### 2.5 Log files

The *chain* and *batch* functions create log files to keep track of all the functions and configuration options that were applied to the data, and the output they generated. The log files also include the random seed, which improves reproducibility of the pipeline (a global random seed can also be set manually). Separate log files are created for each subject/session, and a separate batch log is also created when using the *batch* functions. The subject/session logs are also appended to the preprocessed data, so a processed file will always contain a history of the functions applied to it.

### 2.6 Examples

We use the publicly available multimodal faces dataset (Wakeman and Henson, 2015), v0.1.1 available on OpenfMRI (Poldrack and Gorgolewski, 2017), to illustrate the use of the toolbox. This dataset contains data of 19 subjects, each of which participated in six MEG recording sessions. The subjects engaged in a visual perception task where they saw a series of famous, novel, familiar (repetitions of novel faces) faces, and scrambled faces. To ensure participants were paying attention, participants had to indicate whether faces were symmetrical or asymmetrical with a button press. We analyse these data in a typical analysis workflow that optimally demonstrates the use cases and API of the toolbox; the research question is not based on scientific novelty. Concretely, we first preprocess the MEG data in sensor space, and then reconstruct the sources and combine them into 52 parcels (Kohl et al., 2023). Next, we epoch the parcel time courses, and use a first level GLM to contrast real faces minus scrambled faces, separately for each session. We then model the group effect of this contrast in a second level GLM. All scripts used for this analysis are available on GitHub.^3^ HTML reports and log files are available on OSF.^4^

### 2.7 Development

The toolbox is under active development, and community contributions are welcome on the GitHub page,^5^ in the form of GitHub Issues and pull requests. New osl-ephys versions will be released on GitHub and PiPy when significant changes in the toolbox have been made.

### 2.8 Citing osl-ephys

For the most up to date information on how to cite the toolbox read the CITATION file on GitHub.^6^

## 3 Results

Below, we show the results of using osl-ephys on the multimodal faces dataset with a typical analysis pipeline, including:

1. preprocessing,
2. coregistration and source reconstruction.
3. epoching and first-level GLM.
4. second level (group) statistical analysis using a GLM.

The results shown here are based on osl-ephys 2.1.0, mne 1.3.1, fslpy 3.11.3, and Python 3.8.16.

### 3.1 Batch preprocessing

Listing 2 shows how the preprocessing pipeline is setup in the __main__ body of a Python script (Listing 2), which is necessary in order to use Dask for parallel processing. This first specifies the inputs to preprocessing “batch” function and the config. The config contains a *meta* section specifying the event codes and names for each event, and a *preproc* section with each preprocessing step that will be run in turn. The appropriate function is found by matching the function name to (1) any custom written functions (supplied to the “extra_funcs” parameter), (2) toolbox specific functions, and (3) MNE-Python methods on Raw, and Epochs classes.

**LISTING 2:**
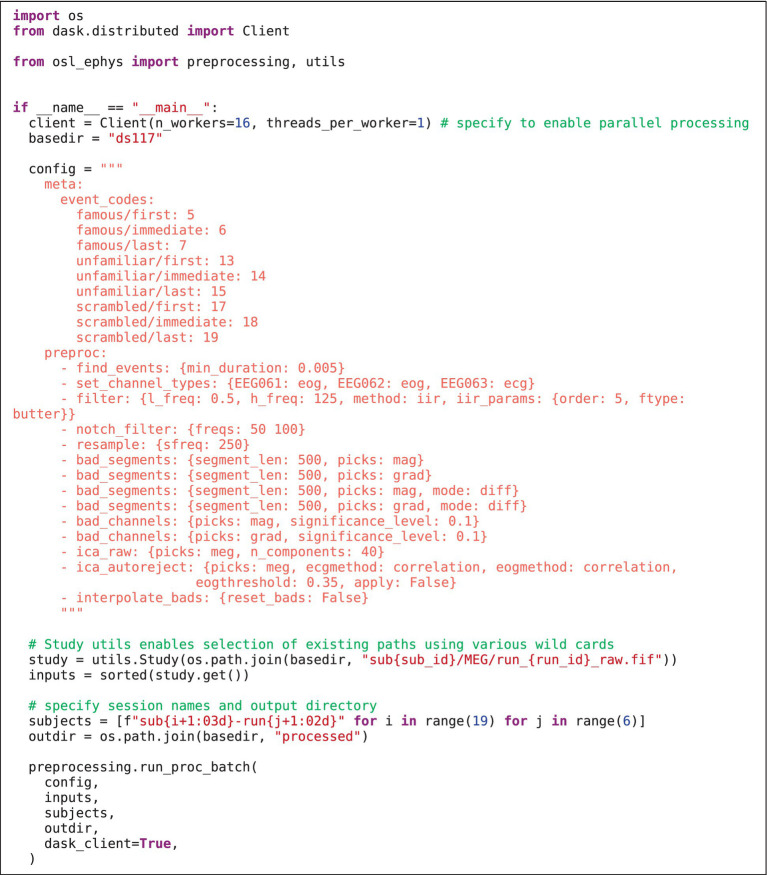
Osl-ephys batch preprocessing script.

**Figure 1:**
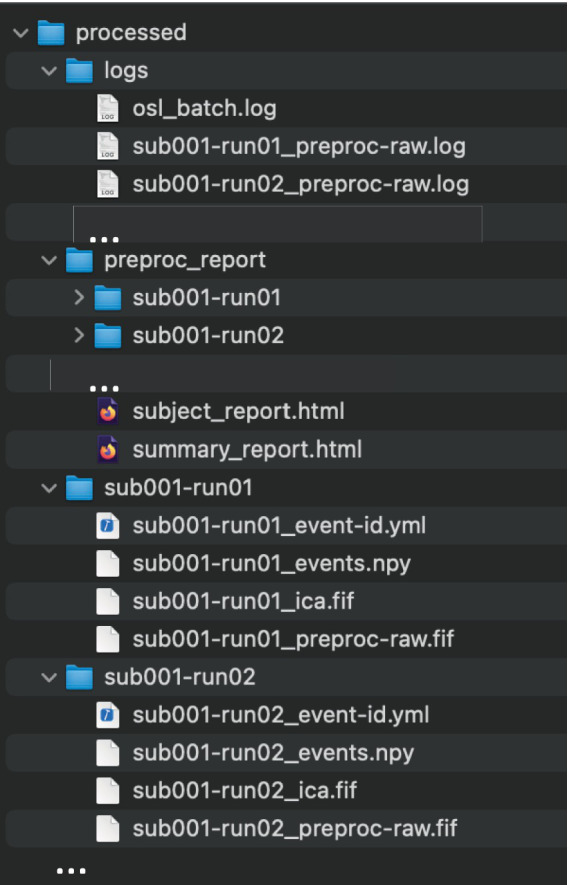
The output directory structure of run_proc_batch. All outputs are saved in the general output directory specified in the function call (“processed”). Within this, a subdirectory is created for each subject/session that contains the preprocessed data, as well as *logs*, and *preproc_report* directories, containing the relevant files for all subjects.

Next, the paths to the raw MEG data are specified using the “Study” class. This enables data paths to be specified using multiple wild cars, and selects existing paths that satisfy the wild cards [here, all paths are selected, but, for example, one can select only the sessions of, e.g., subject 1 using study.get(sub_id = 1)]. An output directory and session subdirectories are then specified for saving each session’s preprocessed data. Lastly, parallel computation is enabled using Dask (having already specified the Dask Client). Running Listing 2 creates the output structure and derivatives in Figure 1. Preprocessing of all 114 files took XX minutes on 12 CPU cores, whereas it took XX times longer (XX minutes) when simply looping the MNE-Python pipeline over sessions on a single CPU.

### 3.2 Logs and preprocessing report

The preprocessing batch function has additionally generated log files for each session and a batch log file. The batch log documents high level information about the batch processing including time stamps, the random seed, the config, and in how many files preprocessing was successful. Each session specific log file documents all processing steps applied to the data, including other relevant function outputs (e.g., the number of bad channels detected). The log files thus provide detailed information and can aid in reproducibility of the pipeline.

**Figure 2:**
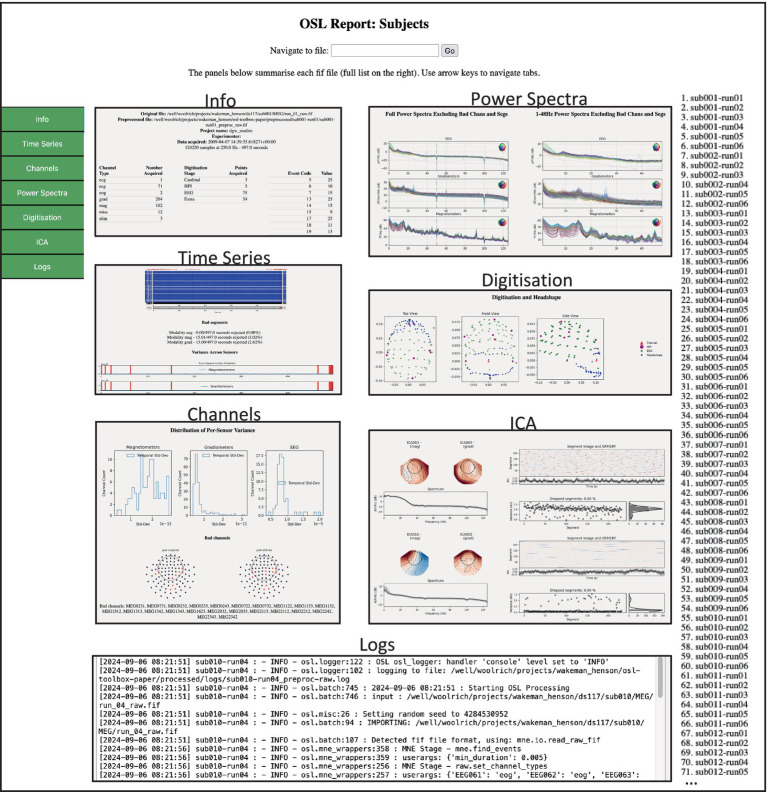
Example of the preprocessing subject report. This HTML page contains tabs for different aspects of QA for each subject/session. The user can browse between tabs on the left for each subject/session in the list on the right. Each tab contains quantitative and qualitative information regarding the preprocessing output.

**Figure 3:**
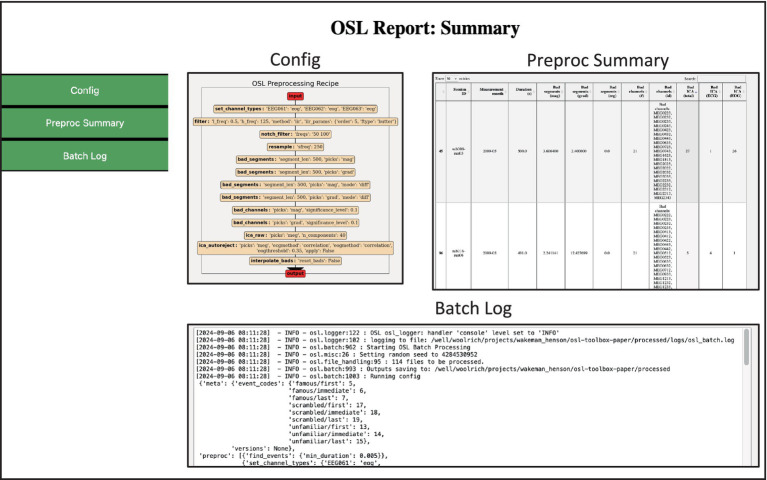
Example of the preprocessing summary report. Contains summary information and quantitative metrics of all files processed. The *Preproc Summary* table is interactive and can guide the user to specific subjects/sessions which might require further attention.

The batch function additionally generates reports that can be used for quality assurance (QA), and which can easily be shared. It contains two HTML files: one for a subject level report (Figure 2) and one for a summary (i.e., group-level) report (Figure 3). The subject report presents detailed qualitative and quantitative metrics of the preprocessing applied to each subject (e.g., general data info, number of bad channels/segments detected, power spectra, etc.), whereas the summary report summarises the batch preprocessing. It includes a table with summary metrics for each subject (e.g., percentage of data marked as “bad,” number of bad channels, number of ECG-related ICs, etc.). This table is interactive and can guide the user to individual subjects/sessions which need to be manually checked. This is especially useful when a large amount of data is processed and manually checking each subject is not feasible.

For example, sorting the table based on *Bad ICA (total)* reveals there were 27 bad ICs detected in *sub008-run03*, of which 26 were labelled EOG. The subject report shows that most of these are spurious, and thus this dataset requires extra attention, for example by changing the preprocessing options or manually adapting the labels (see *3.3 Manual ICA labelling*). The reports also contain the batch and subject logs respectively, and if present, the summary report contains error logs for files that returned errors during preprocessing.

### 3.3 Manual ICA rejection

In MEG preprocessing, ICA is typically used to remove cardiac-and ocular-related artefacts. The ICs that capture these physiological artefacts can potentially be identified using the correlation with the electrocardiogram (ECG) and electrooculogram (EOG) time series, if these were recorded. If they are not, or if they are not of sufficient quality, manual inspection might be necessary. A combination of automatic and manual detection is recommended, i.e., manually refining the automatic first-pass labelling based on the preprocessing report.

During batch preprocessing, ICA is run and identifies ICs with high correlations with the EOG/ECG signals. However, these ICs have not yet removed from the MEG data. As mentioned above, many ICs were spuriously labelled in *sub008-run03*. The user can use the interactive labelling tool to manually correct the selection of bad ICs. It can be called from the command line (in the correct toolbox conda environment) with only a handful required inputs, explained below:

(osle) > osl_ica_label reject_option preproc_dir session_name

- reject_option: indicating which of the ICs should be removed from the data. Can be “all” (i.e., automatically and manually labelled ICs), “manual” (i.e., only manually labelled ICs), or “None” (i.e., save the ICA object but do not remove any components from the data).
- preproc_dir: general output directory, i.e., the same as supplied to the pipeline function.
- session_name: subject/session specific identifier, i.e., the same as supplied to the pipeline function.

In this example, no components have yet been removed from the data. First, the selected components need to be manually checked for a few sessions. Therefore, the command line call is as follows:

(osle) > osl_ica_label None processed sub008-ses03

**Figure 4:**
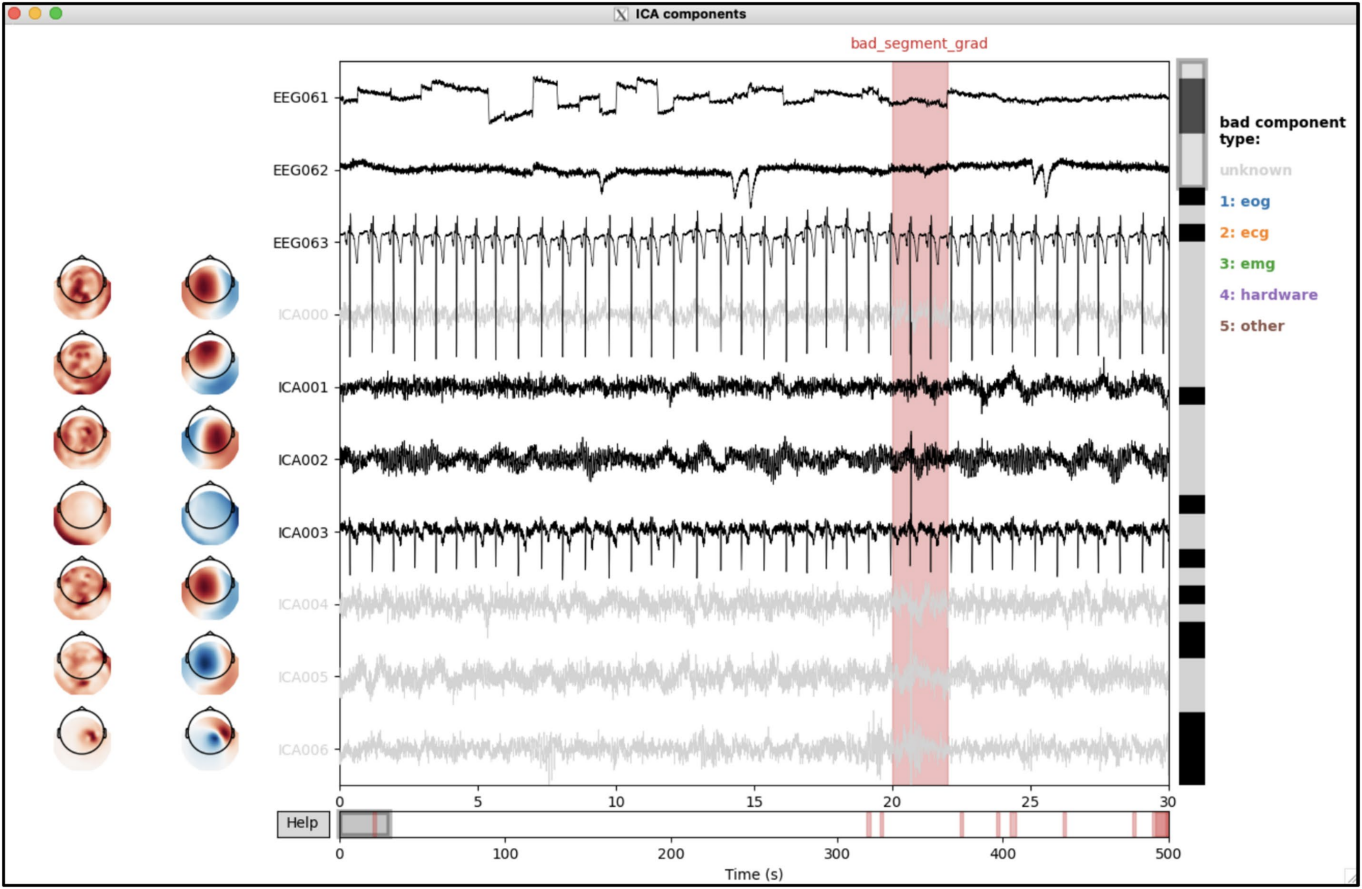
Interactive labelling of independent components (ICs) using the osl_ica_label tool. The weights and time courses for each IC are shown as rows. Bad ICs are indicated as coloured the time courses (i.e., other than black, see types on the right); annotations in the time courses indicate bad segments.

This opens the interactive tool (Figure 4), which shows the IC weights and time courses (and ECG/EOG time courses at the bottom). The user can browse through ICs (vertical scroll bar) and time (horizontal scroll bar) and (de-)select ICs where appropriate, using button pressed to optionally label selected ICs as correlate of artefact types indicated on the right.

Further inspecting the summary and subject report also reveals that the automatic bad IC detection did not identify any EOG-related components and identified spurious ECG-related components in subject 19, and similarly in session 5 of subject 10. The ICA labels of these sessions are also adapted using the same interactive graphical user interface. Once the user is satisfied with the rejected components, the following command line function is used to iteratively remove all the selected components from the data, which will also automatically update the logs and reports:

(osle) > osl_ica_apply processed

### 3.4 Coregistration and source reconstruction

As mentioned before (see section “Source reconstruction”), the toolbox allows to use either outputs from FreeSurfer, or FSL for coregistration and source reconstruction. It also contains wrapper functions to both softwares such that extracting surfaces from sMRI scans can be directly adopted in an osl-ephys pipeline. We here used a pipeline using FSL, since this is a unique feature to this toolbox. Note that examples for using FreeSurfer outputs are available on readthedocs.

**Figure 5:**
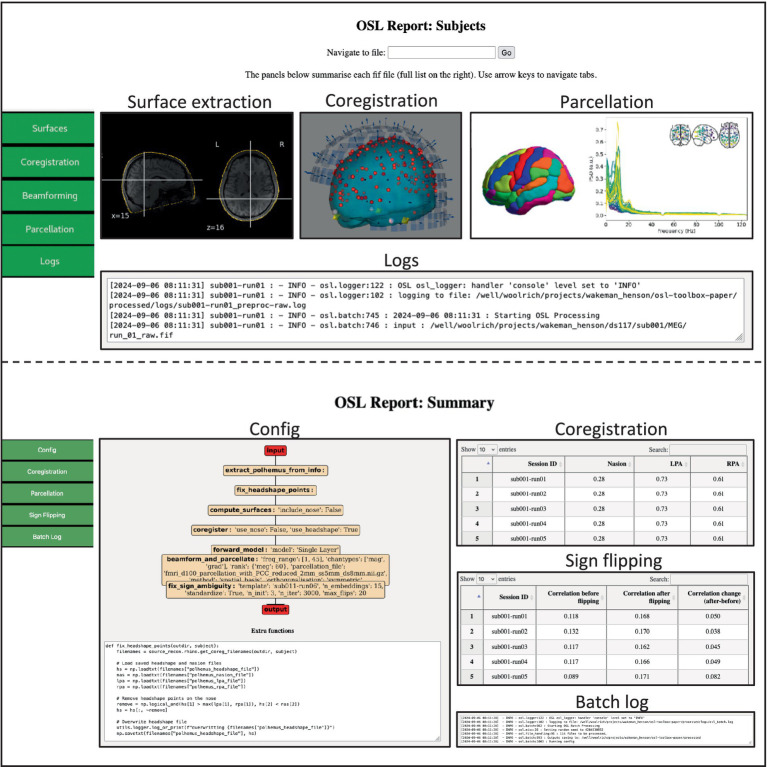
Example of the source_recon subject (top) and summary (bottom) report. The subject report contains figures showing the extracted surfaces, the parcel power spectra, and interactive figures showing the coregistration. The summary report contains summary information and quantitative metrics of all processed files, including interactive tables that can guide the user to specific subjects/sessions which might require further attention.

For coregistration, the digitized head shape (i.e., from Polhemus) is extracted from the preprocessed fif-file and stray points are removed (see Supplementary Listing 1). FSL is used to compute surfaces of the full head surface, including the nose, and of the inside and outside of the skull (i.e., using FLIRT and BET). The coordinate systems of the MEG (device) space, (Polhemus) head space, and the sMRI are coregistered with RHINO (OHBA Analysis Group, 2014), which uses the additional information provided by the nose for coregistration. Then, a forward model is computed using a single shell Boundary Element Model (BEM), and volumetric LCMV beamforming (Van Veen et al., 1997) is used to estimate source activity on an 8 mm volumetric source grid. Source dipoles are combined into a 52 parcels (Kohl et al., 2023) by estimating a spatial basis set over all dipole locations within each parcel, and spatial leakage is reduced between the parcels (Colclough et al., 2015). Lastly, the orientation for each parcel is aligned over subjects (Supplementary Listing 2). This pipeline generates all output data in the same directory structure as the preprocessed data (SI Figure 1), and in addition generates source *subject* and *summary* HTML reports (Figure 5), which can be used for quality assurance.

### 3.5 Epoching and statistical analysis using GLMs

For this example, we compare the activity in each parcel between real faces and scrambled faces (Supplementary Listing 3). We use the versatility of the *preprocessing* batch function to epoch the data around stimulus onset and then run a first-level GLM on each session with regressors for the three different stimulus types (*famous*, *unfamiliar*, and *scrambled* faces) along with a *mean* and *faces vs. scrambled* contrast.

**Figure 6:**
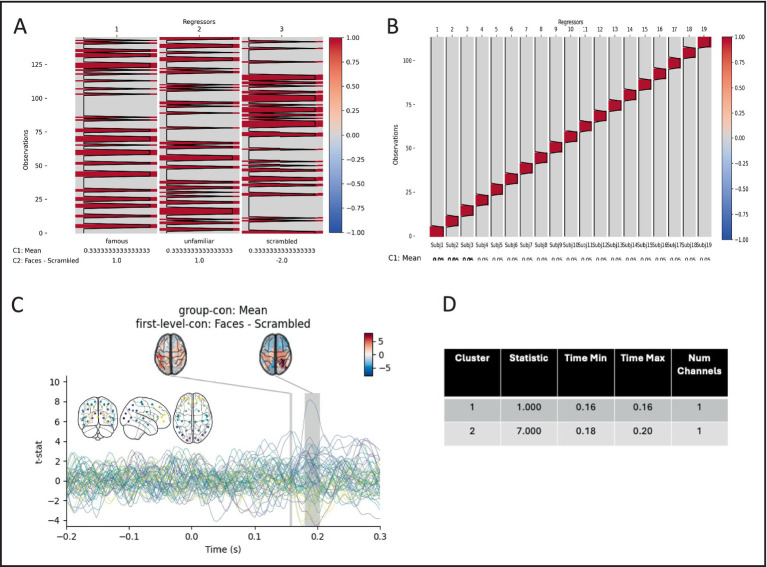
Pictures of real faces elicit statistically different event-related fields than pictures of scrambled faces. **(A)** example of a first level (session) design matrix, with three regressors and two contrasts. **(B)** The second level (group) design contains 19 subject regressors, and one mean contrast. **(C)** The group *Faces – Scrambled* contrast. Coloured lines show individual parcels, with colours in an anterior–posterior gradient (inset). Shaded areas show significant time periods, and topographies the mean t-statistic in each period. **(D)** Extent of significant times periods.

In the same config, we can specify the group (second-level) GLM to be run on the outputs of the first-levels. A design matrix with a regressor for each subject is constructed along with a group mean contrast. Finally, maximum statistic permutation test is used to test whether there is a difference between normal face stimuli, and scrambled faces in the 50–300 ms post stimulus onset window, and significant differences are visualised (Figure 6).

## 4 Discussion

We have presented how the osl-ephys toolbox for the analysis of M/EEG data. This is not a standalone toolbox, but heavily relies on the widely adopted MNE-Python toolbox, FSL and other popular python packages: numpy, scipy, matplotlib, etc. The toolbox aims to augment MNE-Python by providing a config API for reproducible processing of large quantities of data, while providing quality assurance and unique functionalities for data analysis. This includes functions for automatic and manual data preprocessing, FSL-based (Freesurfer independent) volumetric source reconstruction, and statistical analysis, in particular, using GLMs.

Researchers face a number of challenges when analysing M/EEG data. Firstly, analysis is complex and heterogenous. The analysis pipeline depends on the nature and quality of the data, as well as the experimental design and research question. Therefore, analysis flexibility is essential for analysis software. However, analysis complexity and flexibility come with caveats, particularly in terms of transparency and reproducibility. In particular, it is cumbersome and error-prone to manually provide *all* details of an analysis pipeline in the Methods section of academic publications. Even with the growing requirement of funders and journals to provide analysis scripts upon publication of a manuscript, the full details for the analysis pipeline often remain unclear.

Therefore, the toolbox uses a concise and easily shareable “config” API, which reduces the amount of custom written scripts and functions that the researchers need to write (whilst retaining analysis flexibility). In addition, the toolbox keeps track of all processing that took place in log files, and it generates analysis reports that can be used for both reproducibility efforts, and quality assurance.

The high complexity also means that no single analysis toolbox can provide all possible analytical methods, and therefore, the researcher typically needs to stitch together various third-party toolboxes in their analysis pipeline. The toolbox presented here is built on top of the most adopted Python M/EEG analysis toolbox (MNE-Python), and many other Python (and MATLAB) toolboxes contain plugins and/or documentation on how to use their toolbox in combination with MNE-Python. This makes it more straightforward to use different toolboxes. Additionally, the toolbox can be used in a modular fashion, and custom-written and third-party functionality can be easily implemented as an extension to the toolbox by supplying the *chain*/*batch* functions with extra function definitions.

Another challenge is that high analysis complexity means a high entrance barrier for new researchers in the field of M/EEG analysis, and/or programming, especially considering the multidisciplinary nature of the field. This toolbox alleviates this by combining the config API a limited number of functions (in particular the *chain* and *batch* functions) that the user interacts with and taking care of much of the complexity in programming and data bookkeeping on the backend. This is further aided by comprehensive documentation and tutorials. Finally, the analysis reports can also help researchers new to the field, by providing a platform for quality assurance.

In conclusion, the osl-ephys toolbox represents a significant advancement in M/EEG data analysis, offering a balance between flexibility, reproducibility, and ease of use, while addressing key challenges in the field and paving the way for more accessible and robust neuroimaging research.

## Data Availability

The datasets presented in this study can be found in online repositories. The names of the repository/repositories and accession number(s) can be found at: https://osf.io/2rnyg/; https://openfmri.org/dataset/ds000117/; https://github.com/OHBA-analysis/osl-ephys/tree/main.

## Footnotes

^1^ https://osl-ephys.readthedocs.io/en/latest/

^2^ https://github.com/OHBA-analysis/osl-ephys/tree/main

^3^ https://github.com/OHBA-analysis/osl-ephys/tree/main/examples/toolbox-paper

^4^ https://osf.io/2rnyg/

^5^ https://github.com/OHBA-analysis/osl-ephys/tree/main

^6^ https://github.com/OHBA-analysis/osl-ephys/blob/main/CITATION.cff
