# Supplementary material for: osl-ephys: a Python toolbox for the analysis of electrophysiology data
Source: Front Neurosci. 2025 Feb 21;19:1522675. doi: 10.3389/fnins.2025.1522675 (PMC11885225; doi:10.3389/fnins.2025.1522675)
Supplement: Supplementary file 1 [file Data_Sheet_1.docx]

osl-ephys: A Python toolbox for the analysis of electrophysiology data – Supplemental Information

Mats W.J. van Es, Chetan Gohil, Andrew J. Quinn, Mark W. Woolrich

Listing 1. Example coregistration and source reconstruction script.

**import** os

**import** numpy **as** np

**from** dask.distributed **import** Client

**from** osl_ephys **import** source_recon, utils

source_recon.setup_fsl(”~/fsl") # FSL needs to be installed

**def** fix_headshape_points(outdir, subject):

filenames = source_recon.rhino.get_coreg_filenames(outdir, subject)

# Load saved headshape and nasion files

hs = np.loadtxt(filenames["polhemus_headshape_file"])

nas = np.loadtxt(filenames["polhemus_nasion_file"])

lpa = np.loadtxt(filenames["polhemus_lpa_file"])

rpa = np.loadtxt(filenames["polhemus_rpa_file"])

# Remove headshape points on the nose

remove = np.logical_and(hs[1] > max(lpa[1], rpa[1]), hs[2] < nas[2])

hs = hs[:, ~remove]

# Overwrite headshape file

utils.logger.log_or_print(f"overwritting {filenames['polhemus_headshape_file']}")

np.savetxt(filenames["polhemus_headshape_file"], hs)

**if** __name__ == "__main__":

utils.logger.set_up(level="INFO")

client = Client(n_workers=16, threads_per_worker=1)

config = """

source_recon:

- extract_polhemus_from_info: {}

- fix_headshape_points: {}

- compute_surfaces:

include_nose: False

- coregister:

use_nose: False

use_headshape: True

- forward_model:

model: Single Layer

- beamform_and_parcellate:

freq_range: [1, 45]

chantypes: [mag, grad]

rank: {meg: 60}

parcellation_file: Glasser52_binary_space-MNI152NLin6_res-8x8x8.nii.gz

method: spatial_basis

orthogonalisation: symmetric

"""

basedir = "ds117"

proc_dir = os.path.join(basedir, "processed")

# Define inputs

subjects = [f"sub{i+1:03d}-run{j+1:02d}" **for** i **in** range(19) **for** j **in** range(6)]

preproc_files = sorted(utils.Study(os.path.join(proc_dir, "sub{sub_id}- run{run_id}/sub{sub_id}-run{run_id}_preproc-raw.fif")).get())

smri_files = np.concatenate([[smri_file]*6 **for** smri_file **in** sorted(utils.Study(os.path.join(basedir, "sub{sub_id}/anatomy/highres001.nii.gz"))).get()])

# Run source batch

source_recon.run_src_batch(

config,

outdir=proc_dir,

subjects=subjects,

preproc_files=preproc_files,

smri_files=smri_files,

extra_funcs=[fix_headshape_points],

dask_client=**True**,

)

**import** os

**from** glob **import** glob

**from** dask.distributed **import** Client

**from** osl_ephys **import** source_recon, utils

source_recon.setup_fsl("~/fsl")

# Directory containing source reconstructed data

proc_dir = "ds117/processed"

src_files = sorted(utils.Study(os.path.join(proc_dir,
"sub{sub_id}-run{run_id}/parc/parc-raw.fif")).get())

**if** __name__ == "__main__":

utils.logger.set_up(level="INFO")

subjects = [f"sub{i+1:03d}-run{j+1:02d}" **for** i **in** range(19) **for** j **in** range(6)]

# Find a good template subject to match others to

template = source_recon.find_template_subject(

proc_dir, subjects, n_embeddings=15, standardize=**True**,

)

# Settings

config = f"""

source_recon:

- fix_sign_ambiguity:

template: {template}

n_embeddings: 15

standardize: True

n_init: 3

n_iter: 3000

max_flips: 20

"""

# Setup parallel processing

client = Client(n_workers=16, threads_per_worker=1)

# Run sign flipping

source_recon.run_src_batch(config, proc_dir, subjects, dask_client=**True**)

Listing 2. Example sign-flipping script.


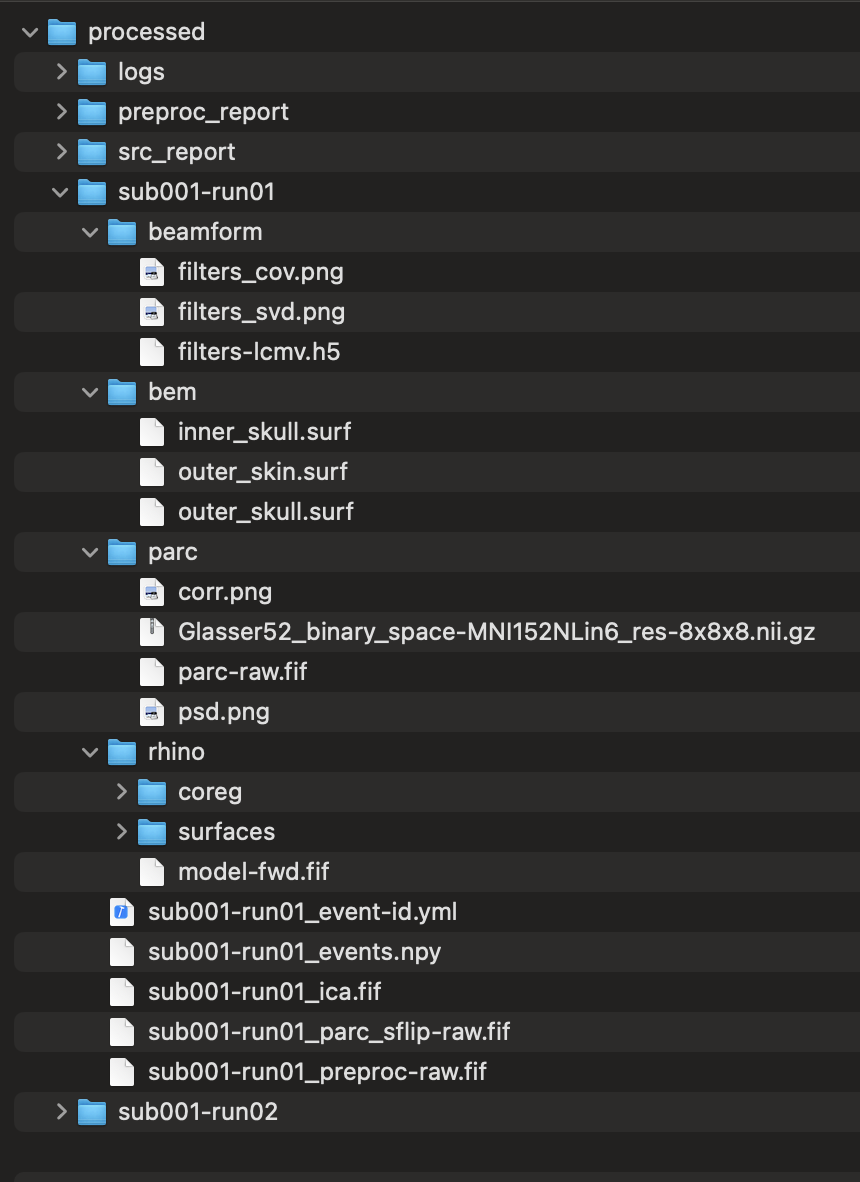


…

Figure 1. The output directory structure of the source_recon batch function. All outputs are saved in the same general directory and subject/session directories that contain the previously preprocessed data.

**import** os

**import** numpy **as** np

**import** glmtools

**import** matplotlib.pyplot **as** plt

**from** dask.distributed **import** Client

**from** osl_ephys **import** preprocessing, glm

**if** __name__ == "__main__":

client = Client(n_workers=16, threads_per_worker=1)

config = """

preproc:

- read_dataset: {ftype: sflip_parc-raw}

- epochs: {picks: misc, tmin: -0.2, tmax: 0.3}

- glm_add_regressor: {name: famous, rtype: Categorical, codes: [5 6 7]}

- glm_add_regressor: {name: unfamiliar, rtype: Categorical, codes: [13 14 15]}

- glm_add_regressor: {name: scrambled, rtype: Categorical, codes: [17 18 19]}

- glm_add_contrast: {name: Mean, values: {famous: 1/3, unfamiliar: 1/3, scrambled: 1/3}}

- glm_add_contrast: {name: Faces-Scrambled, values: {famous: 1, unfamiliar: 1, scrambled: -2}}

- glm_fit: {target: epochs, method: glm_epochs}

group:

- glm_add_regressor: {name: Subject, rtype: Categorical, key: Subject, codes: unique}

- glm_add_contrast: {name: Mean, values: unique, key: Subject}

- glm_fit: {method: epochs, tmin: 0.05, tmax: 0.3}

- glm_permutations: {method: epochs, target: group_glm, contrast: Mean, type: max, nperms: 1000, threshold: 0.99}

"""

proc_dir = "ds117/processed"

src_files = sorted(utils.Study(os.path.join(proc_dir,
"sub{sub_id}-run{run_id}", "sub{sub_id}-run{run_id}_sflip_parc-raw.fif")).get())

subjects = [f"sub{i+1:03d}-run{j+1:02d}" **for** i **in** range(19) **for** j **in** range(6)]
 covs = [f"Subject": [sub.split("-")[0]**for** sub **in** subjects]

preprocessing.run_proc_batch(

config,

src_files,

subjects,

outdir=proc_dir,

ftype='raw',

covs=covs,

dask_client=**True**,

overwrite=**True**,

gen_report=**False**,

skip_save=['events', 'raw', 'ica', 'event_id', 'sflip_parc-raw'],

)

Listing 3. An example script for epoching, first and second level GLM, and permutation testing.
